# SEDA: A software package for the Statistical Earthquake Data Analysis

**DOI:** 10.1038/srep44171

**Published:** 2017-03-14

**Authors:** A. M. Lombardi

**Affiliations:** 1 Istituto Nazionale di Geofisica e Vulcanologia, Via di Vigna Murata 605, 00143 Rome, Italy

## Abstract

In this paper, the first version of the software SEDA (SEDAv1.0), designed to help seismologists statistically analyze earthquake data, is presented. The package consists of a user-friendly Matlab-based interface, which allows the user to easily interact with the application, and a computational core of Fortran codes, to guarantee the maximum speed. The primary factor driving the development of SEDA is to guarantee the research reproducibility, which is a growing movement among scientists and highly recommended by the most important scientific journals. SEDAv1.0 is mainly devoted to produce accurate and fast outputs. Less care has been taken for the graphic appeal, which will be improved in the future. The main part of SEDAv1.0 is devoted to the ETAS modeling. SEDAv1.0 contains a set of consistent tools on ETAS, allowing the estimation of parameters, the testing of model on data, the simulation of catalogs, the identification of sequences and forecasts calculation. The peculiarities of routines inside SEDAv1.0 are discussed in this paper. More specific details on the software are presented in the manual accompanying the program package.

This paper illustrates the capabilities of the first version of *SEDA (Statistical Earthquake Data Analysis, SEDAv1.0*), a new software designed for the statistical analysis of earthquake data.

The tools collected in SEDAv1.0 are classified in two main topics: Catalog Analysis, for the descriptive analysis of an earthquake catalog and for the selection of its subsets, and ETAS Model, allowing the analysis of an earthquake database by the ETAS (Epidemic Type Aftershock Sequence) modeling^1^,^2^.

The first class of tools call original, but not innovative, codes. These are implemented to give a support to the user, in managing the database and in evaluating its homogeneity and magnitude completeness.

The second class of tools is the core of SEDAv1.0 and contains original and partially innovative Fortran codes for the ETAS modeling. To the knowledge of the author, this is the first time that such a comprehensive set of tools, based on ETAS, is collected in a single free software. Some packages have been developed and made available in the past, but they refer to different versions of the ETAS model and allow only a partial analysis of an earthquake catalog. A list of websites from which each package may be downloaded is reported in Table 1, together with a short description and some references.

SEDA has been developed in order to guarantee reproducibility of published research results, which has become a prominent issue in several academic fields^3^,^4^ and is recommended by the most important scientific journals that promote open science^5^. Actually, the computational methods that are behind many published papers are often not fully explained or described, both due to constraints imposed by the traditional research papers and to the reluctance to share intellectual property. As a consequence, research results are difficult to reproduce, either for verifying their correctness or for building on them in future research and applications. A way to guarantee research reproducibility is sharing the codes used in scientific papers. This means that the source or the executable code used to produce the results is freely accessible to the public. This allows replication of results and ensures that the scientific community can apply the methodology to their own data, without the need of re-implementing the algorithms. Moreover, making the software available allows the evaluation of its performance on any dataset, leading to better quality of codes themselves, and the comparison of different methodologies^5^,^6^,^7^,^8^.

The main aim of this paper is illustrating the capabilities and peculiarities of SEDAv1.0 and describing in details the algorithms and methodologies implemented inside it. Anyway, technical details on the software use are presented in the manual accompanying the program package.

## Program Description

SEDAv1.0 was created using GUIDE, the graphical user interface development environment of Matlab (http://www.mathworks.com). Its purpose is to allow an easy interaction (mainly input file preparation and output files display) with the Fortran codes, created for the numerical computations.

SEDAv1.0 has two versions of the most expensive Fortran codes: one, faster, parallel and one, slower, non-parallel version. The Fortran codes have been compiled by Gfortran (http://gcc.gnu.org/wiki/GFortran or http://hpc.sourceforge.net). The parallel versions have been compiled by using the Mpich Fortran library (www.mpich.org), a freely available, portable implementation of MPI (Message Passing Interface), largely used in parallel computing.

SEDAv1.0 is mainly devoted to produce accurate, fast (as far as possible) and comprehensive outputs, which are all saved in ASCII files. Less care has been taken for the graphic appeal, which will be improved in the future. Anyway, the software is rich in maps and plots, to help the user to more easily understand the results. All graphics are created using Matlab graphic capabilities. Specifically, the maps are generated by means of M_Map, a free mapping package for Matlab, written by Rich Pawlowicz (www.eoas.ubc.ca/rich/map.html). The M_Map routines are already included in SEDAv1.0, into the directory *m_map*, together with the GSHHS coastline databases, downloaded from the website http://www.ngdc.noaa.gov/mgg/shorelines/data/gshhs.

When the program starts the main graphical user interface (GUI) is displayed. From this main GUI, the user can choose a class of tools, *“Catalog Analysis”* or *“ETAS model”*, described in the following sections. Table 2 is a summary of routines implemented in SEADv1.0; for each of them information about the existence of parallel/non-parallel versions is reported.

The first version of SEDA runs under MAC, but the next version will include a version for Windows computers. SEDAv1.0 is freely provided via the Zenodo open access platform (https://zenodo.org), a service that allows deposit and DOI assignment to software, besides ensuring an easy and stable access. Please, refer to https://zenodo.org/record/55277 to download the SEDAv1.0 software package for MAC.

I have tried to make SEDAv1.0 versatile, user-friendly, and as accurate as possible. The software has been tested, but there could be undetected errors. Considerable effort and time have been put in developing and testing all tools of SEDAv1.0. Whenever possible, analytical results have been validated with experimental data. Nevertheless, some bugs remain and some features may remain cryptic to many users. If you have questions, suggestions or bug to report, please, send an e-mail at the address annamaria.lombardi@ingv.it.

## Catalog Analysis

This part of the package is devoted to a quick descriptive analysis and to subsets selection of an earthquake catalog. The main goal of this class of tools is to allow the user to select the data useful to his/her analysis. The tools implemented in the first version of SEDA are

1. *“Select a sub catalog”*: to select and to save a sub catalog of a loaded database, by giving temporal, magnitude or spacing criteria; the type of spatial selection may be rectangular or circular;
2. *“B-value Analysis”*: to make a completeness magnitude and b-value analysis of a catalog;
3. *“Figures”*: to plot some descriptive figures (the cumulative distribution of events in time, the time-magnitude plot, the time-longitude-latitude-depth plots, a map of the events).

In SEDAv1.0 only the Gutenberg-Richer Law is implemented for the magnitude distribution. The probability density function is


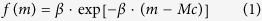


where *β* = *b* · *ln(10*) is a parameter and *Mc* is the completeness magnitude of the database.

**Figure 1:**
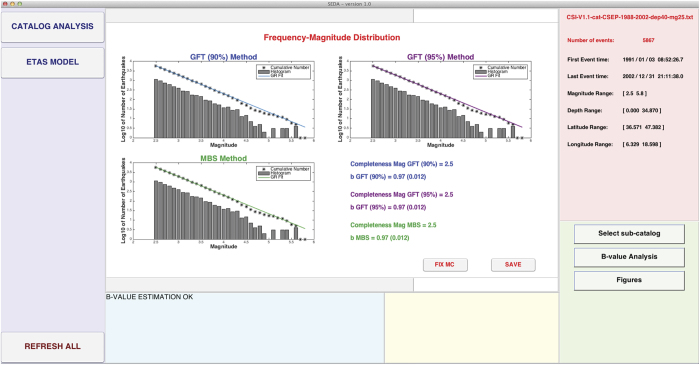
Screenshot of SEDAv1.0 showing the results of the Completeness Magnitude and B-value Analysis on CSI-1.1 Italian Catalog (see text for details).

The system assumes a magnitude step of 0.1 and uses two methods to estimate *b* and *Mc* (Fig. 1):

1. The Mc and B-value Stability method (MBS)^16^,^17^;
2. The Goodness of Fit Test method (GFT)^18^.

The GFT method is performed both at 90% and at 95% confidence levels. Moreover, SEDAv1.0 fixes a magnitude range equal to 0.5 to calculate the b-value means, needed to apply the MBS method^17^. All these limits will be relaxed and new estimation methods and further magnitude distributions will be introduced in the future.

## ETAS (Epidemic Type Aftershocks Sequence) Model

This part of the package provides some tools concerning both the Time-Magnitude (TM) and the Time-Magnitude-Space (TMS) ETAS modeling^1^,^2^,^4^.

The conditional intensities of the TM and TMS ETAS models, implemented in SEDAv1.0 are, respectively:


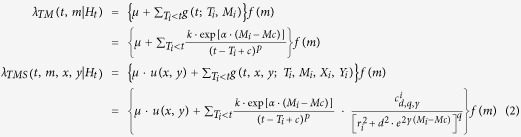


where

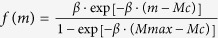
 is the magnitude probability density function and *Mmax* is the maximum magnitude allowed;
2. *H*_*t*_ is the history of the process up the time *t;*
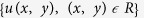
is the spatial density function of the background events in the region *R* of interest. This is assumed uniform in each of the *N*_*c*_ cells *C*_*j*_ (of area *A*_*j*_) of a regular grid (in degrees), covering *R*, so that

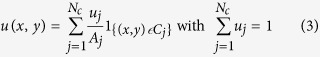

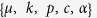
 and 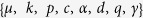 are the parameters, to be estimated, for the TM and TMS model, respectively;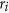 is the distance (in kms) between the location (*x, y*) and the epicenter of the *i*-th event (*X*_*i*_*, Y*_*i*_);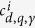 is a normalization constant so that
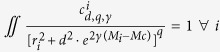
.

The background grid defines the region of interest *R*. Possible events of the catalog occurring outside *R* are included into calculations only for the possible triggering interactions with the events inside *R*. In this way, the background grid defines the target region *R*, for which the ETAS model is estimated, whereas a larger learning region, used to set the interaction rules more correctly, is the area covered by the catalog.

The period for which the ETAS model is estimated or applied is called target period. The seismicity in this period may be affected by the triggering effect of previous earthquakes, so SEDAv1.0 offers the opportunity to include a precursory (or learning) period. In this way the triggering effect of the precursory earthquakes on the target period is taken into account.

Because of memory problems in some FORTRAN codes, all the ETAS tools may be run on catalogs with less than 12,000 events above *Mc* and background grids with *N*_*c*_ = 100,000 cells at most. The first restriction will be relaxed in a later version of SEDA. The second should not undermine the ability of SEDAv1.0, even for very large regions.

SEDAv1.0 collects two sets of ETAS tools, described below:

1. The *ETAS Basic tools*, for the main operations supported by an ETAS model;
2. The *ETAS Additional tools*, to deep the investigation of a catalog, by means of ETAS modeling.

### ETAS Basic Tools

This section describes the theoretical background and some applicative aspects of the ETAS Basic Tools, which are all you need to analyze an earthquake catalog by the ETAS model. All Figures cited in this section refer to the example described in the following.

The available ETAS Basic Tools in SEDAv1.0 are:

- *“Estimation of Parameters”*: to estimate the ETAS models on earthquake catalogs;
- “Log-likelihood *Calculation”*: to compute the Log-Likelihood of an ETAS model on a seismic catalog;
- *“Declustering”*: to decluster a catalog by means of an ETAS model;
- *“Testing the model”*: to test a version of an ETAS model on data;
- *“Forecasting”*: to do forecasting calculations by means of ETAS modeling;
- *“Simulation”*: to simulate earthquake catalogs by ETAS models.

The following subsections provide a description of each tool.

#### Estimation of Parameters

This tool allows the Maximum Likelihood Estimation (MLE) of a TM or TMS ETAS model on an imported catalog. The algorithm implemented in SEDAv1.0 is an innovative method based on Simulated Annealing (SA)^19^. Unlike the Newton methods, largely used by the other published codes^9^,^11^, the method implemented here allows a suitable evaluation of the model uncertainties, by means of multiple runs. These provide a probability distribution for each parameter and for the background spatial distribution. Moreover, the comparison of parameter values found in different runs helps to detect possible correlations among them and, therefore, a multimodal distribution of the maximum log-likelihood. Finally, the SA method performs a systematic setting of the model, without any dependence on the starting values of parameters, thereby avoiding the risk of finding a local maximum of the log-likelihood^19^.

SA is a stochastic method to solve problems of multidimensional global optimization, i.e. problems with the following form


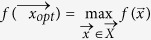


where 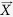 is a *D*-dimensional subset of *R*^*D*^.

SA algorithms are random iterative procedures that generate a candidate point 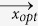 and move to this point or stay at the current one based on a stochastic mechanism. The latter is controlled by the temperature *T*; when decreased, the search becomes more directive.

The specific problem consists in finding the set of parameters 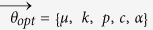
or 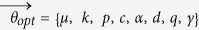, for the TM and the TMS ETAS model respectively, that maximize the log-likelihood 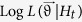. The explicit expression of the log-likelihood is reported in the next subsection. As shown below, the log-likelihood computation allows also the evaluation of the expected number of target events 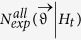 (i.e. the events occurred in the target period and, for the TMS model, in the region *R*). More formally, the SA algorithm for the MLE of an ETAS model can be described as follows.

1. *Initialization.* Generate an initial random solution 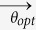. Select a value for the initial temperature *T*_*0*_ > *0*. Set the count *j* = *0*.

2. *Inner loop.* Set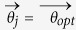 and repeat the following *N*_*in*_ times:

  2a. Generate the next candidate 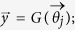


  2b. Sample a uniformly distributed random number *p*∈ [*0, 1*] and set


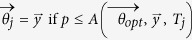


where *A* is a suitable acceptance function;

  2c. Set 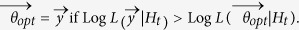


3. *Outer loop.* Check a stopping criterion and, if satisfied, then STOP; otherwise

  3a. Set 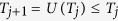 and *j* *=* *j* + *1*;

  3b. Go back to the Inner loop.

The only difference between the algorithm implemented in SEDAv1.0 and the method presented in Lombardi^19^, is a new cooling schedule *U* for the temperature. Specifically, SEDAv1.0 adopts the schedule proposed by Ingber^20^
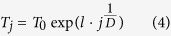


where:

- *T*_*j*_ is the temperature at the *j*-th iteration and *T*_*0*_ is the initial temperature;
- *D* is the number of parameters (5 for TM and 8 for TMS ETAS models);
- *l* has the form *l* = *−δ* · *exp*(−*ν/D*).

By imposing that after *J* = *exp(ν*) iterations the values of the temperature is 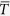, one may estimate the parameters *δ* and *ν* by the formulas


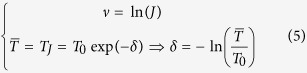


By applying the algorithm on simulated data, with *J* varying from *10* to *100* and 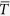 varying from *10*^−*8*^ to *10*^−*4*^, I find that the faster algorithm is obtained with *J* = *30* and 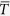 *=* *10*^−*5*^, giving *δ* *=* *3.4* and *ν* *=* *13.8*. So that the cooling schedule adopted in SEDAv1.0 is


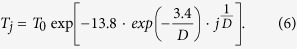


SEDAv1.0 gives two options for including the background inside the TMS model:

1. To estimate both the overall rate *μ* and the spatial PDF *u(x, y*) of the background seismicity;
2. To import a grid representing the spatial distribution of the background 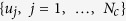; in this way the code estimates the overall background rate *μ*, without estimating the background spatial probability distribution *u(x, y*).

If the first option is chosen, the background spatial distribution *u(x, y*) is estimated by the kernel method proposed by Zhuang *et al*.^11^. Further details on the algorithm may be found in Lombardi^19^.

To limit the running time, SEDAv1.0 automatically assigns the number of runs by following the rule


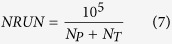


where *N*_*P*_ and *N*_*T*_ are the number of precursory and target events of the catalog, respectively. Anyway, you may set the value of *NRUN* by a specific edit box.

**Figure 2:**
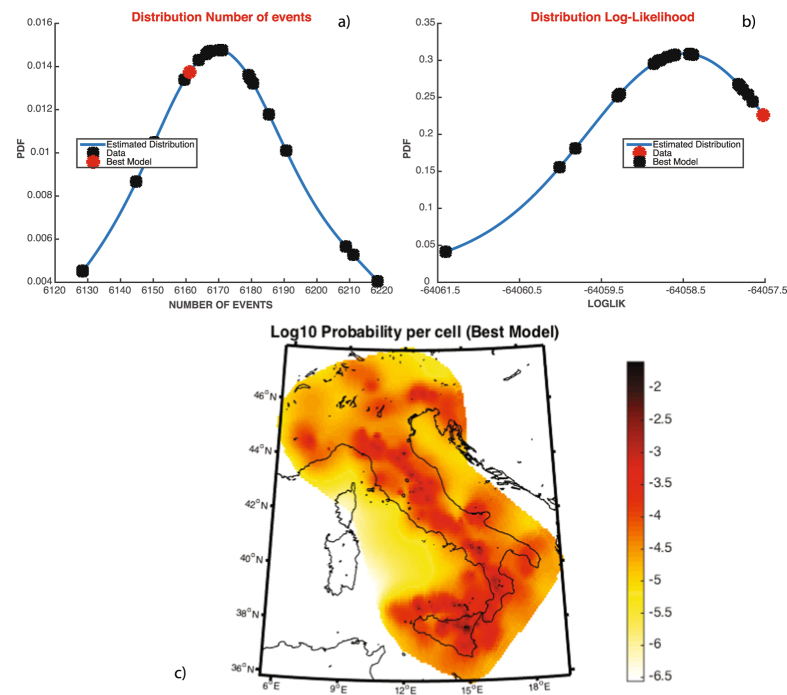
Results of the Estimation of the TMS ETAS model on the CSI-1.1 Italian Catalog. All panels are provided by SEDAv1.0. (**a**) Distribution of the number of events expected by the best ETAS model obtained in each run of the Simulated Annealing algorithm; the red star marks the observed number of events of the CSI-1.1 catalog. (**b**) The same of (**a**) but for the Log-likelihood value. (**c**) Map of the background probabilities {u_i_, i = 1,.., N_c_}. The map is generated by means of M_Map, the free mapping package for Matlab, written by Rich Pawlowicz (www.eoas.ubc.ca/rich/map.html).

A summary of results will appear on the man GUI of SEDAv1.0 (Fig. 2) and specifically:

1. The parameters 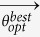 together with the 
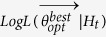
 and the 
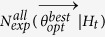
 for the “best” model, i.e. the model with the maximum Log-Likelihood, between the values obtained from all *NRUN* runs;
2. The median and the 95% confidence bounds for each parameter (including the background probabilities *u*_*i*_), for the maximum Log-Likelihood and for the expected number of target events, all inferred by the *NRUN* values estimated.

**Figure 3:**
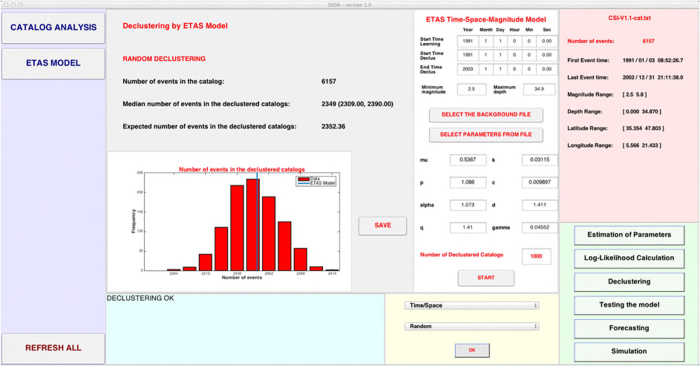
Screenshot of SEDAv1.0 showing the Results obtained by applying the Random Declustering algorithm of SEDAv1.0 on the CSI-1.1 catalog. The histogram refers to the sizes of all 1000 declustered catalogs.

Finally, you can display some figures, by clicking on the appropriate icons (Fig. 3):

1. The plot of the probability density distribution of the expected number of target events, of Log-Likelihood and of each parameter, obtained by the *NRUN* models;
2. The plot of all *NRUN* couples of parameter values, to show possible correlations among them;
3. (Only for the TMS model) the map of the background probabilities *u*_*j*_ for the “best” model or for a percentile of the *NRUN* estimated models;

All the probability density distributions are estimated by applying a normal kernel smoothing method. The *NRUN* sets of parameters (including the grids of background probabilities *u*_*j*_) are saved in files (see the User Manual of SEDAv1.0 for details).

The Additional ETAS tools “*Analysis of parameters*” and “*Background map*” allow to load again the output files generated by the “*Estimation of parameters*” tool.

#### Log-Likelihood Calculation

This tool allows the Log-Likelihood computation for a TM or TMS ETAS model on an earthquake catalog, given a set of parameters (including the spatial background distribution for the TMS model). The formulas used for the Log-Likelihood of the TM and TMS ETAS models are, respectively:


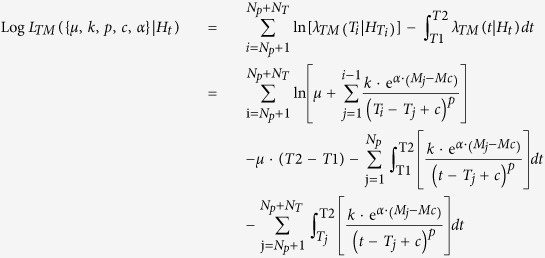


and


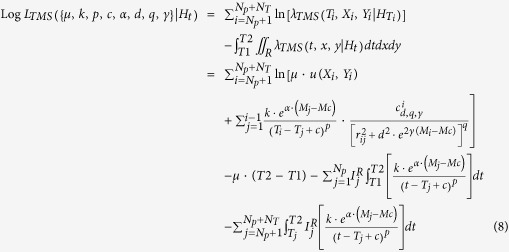


where:

1. *N*_*P*_ and *N*_*T*_ are the number of observed precursory and target events, respectively;
2. *T1* and *T2* are the starting and ending time of the target period;
3. *H*_*t*_ is the history of observations up to the time *t*, including the precursory events;
4. *R* is the region of interest, defined from the background grid;

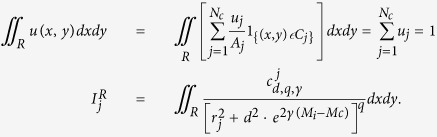


The second term of the log-likelihood, the integrals 
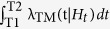
 and 
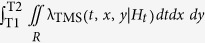
, represents the overall expected number of target events 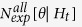 with magnitude above *Mc* and, for TMS models, in the region *R*.

#### Declustering

This tool allows the declustering of an earthquake catalog, i.e. the identification of the background seismicity, by using a TM or TMS ETAS model. The procedure consists in assigning the probabilities 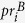
of being a background event to all target events of the catalog. Then, the system identifies the background events, based on the computed probabilities.

The system computes the background probabilities 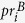 by the formulas


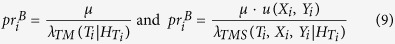


for the TM and TMS ETAS model, respectively.

A histogram of background probabilities 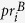 is displayed by the ETAS Additional tool *“Trigg/Back Probabilities”*, which allows estimating, separating and visualizing the contribution of the background and triggered seismicity.

To identify the background events, the user may choose one of two declustering methods and specifically:

1. The *Fixed Threshold Method* that selects all the events with a background probability larger than a prefixed threshold (chosen by the operator);
2. The *Random Method* that applies the algorithm of Zhuang *et al*.^11^ and identifies the background events by a random procedure. In this case, the system generates *NCAT* (potentially different) declustered catalogs.

The first method identifies the events with large 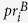 (above a fixed threshold) as background. It may give a bias between the expected and the observed number of background events, i.e 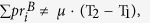 and makes a deterministic classification of the events in background and triggered. Moreover, the resulting background might fail the hypothesis of Poissonian distribution, assumed by the ETAS modeling. The second method is more correct by a theoretical point of view, since it makes a probabilistic treatment, based on the ETAS model, and includes the uncertainties in the assignment of events to background.

A summary of results will appear on the man GUI to compare the expected number of background events with what obtained by applying the SEDAv1.0 tools (Fig. 3).

#### Testing the Model

**Figure 4:**
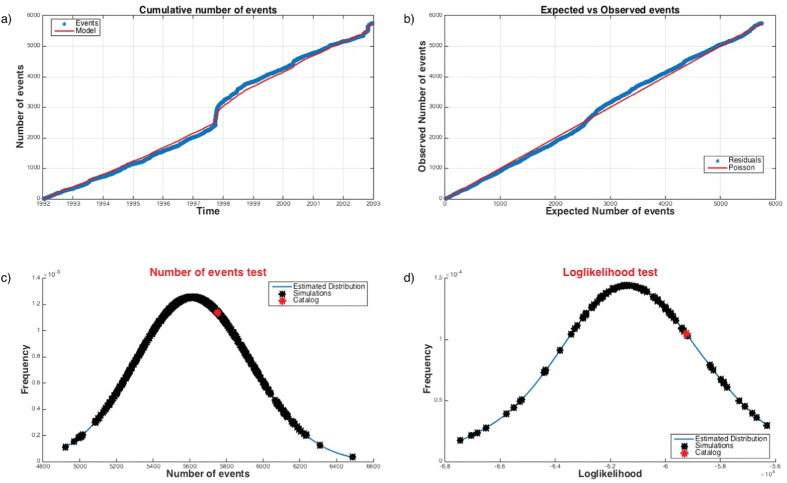
Results of the test of the ETAS TMS model for the CSI-1.1 catalog. (**a**) Comparison of the observed (red line) and expected (blue line) cumulative numbers of events vs the time. (**b**) Residual Analysis: comparison of the observed and expected number of events. (**c**) Number of events test: expected distribution of the number of events from the TMS ETAS model; the red star marks the observed number of events in the CSI-1.1 catalog. (**d**) The same of (**c**) but for the Log-likelihood test. The map is generated by means of M_Map, the free mapping package for Matlab, written by Rich Pawlowicz (www.eoas.ubc.ca/rich/map.html).

This tool allows you to test a given TM or TMS ETAS model on an earthquake catalog. Actually, SEDAv1.0 has three options: the *Residuals Analysis*, the *Number of events test* and the Log-Likelihood test (Fig. 4).

The *Residuals Analysis* is a well-known procedure based on the transformation of the time axis *t* (days) into a new scale *τ*^1^,^2^, using the functions:


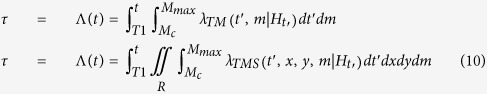


for the TM and TMS models, respectively.

The random variable τ represents the expected number of occurrences in the time period [*T1, t*] and in the whole region *R* (for TMS models), with a magnitude above *Mc*. If the ETAS model well describes the temporal evolution of the process, the transformed data *τ*_*i*_ = *Λ(T*_*i*_), usually called residuals, are expected to behave like a stationary Poisson process with a unit rate. SEDAv1.0 tests this hypothesis by the one-sample Kolmogorov-Smirnov (KS1) test, which determines if the inter-event transformed times follow an exponential distribution, and the RUNS test, which examines whether there is a temporal trend in the inter-event transformed times.

A summary of results will appear on the man GUI (Fig. 4) and specifically:

1. The p-values of the KS1 and RUNS tests for residuals;
2. The comparison of the overall, background, triggered expected and observed numbers of events, in the target period;
3. The plot of the expected vs the observed number of events (Fig. 4b);
4. The plot of cumulated distributions in time for overall, background, triggered expected and observed numbers of events (Fig. 4a).

The “observed” number of background and triggered events (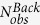 and 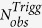) are computed as the sum of probabilities 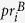 (see eq. 9) and 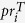 = 1 − 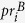, respectively:


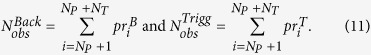


These formulas give 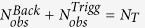, that is the observed number of target events.

The expected overall, background and triggered numbers of events (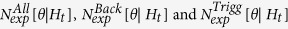) are computed by integrating the related rates in time, magnitude and, for the TMS model, space. Specifically, you have for the TM model:


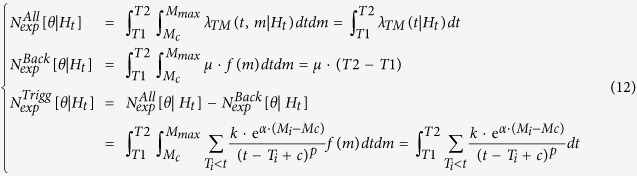


and for the TMS model:


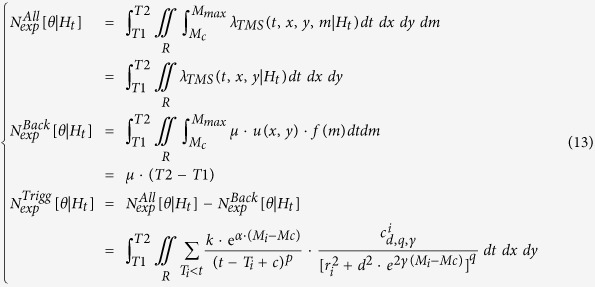


All the variables provided by this tool are computed also by the ETAS Additional tool *“Trigg/Back Probabilities”*, which allows estimating, separating and visualizing the contribution of the background and triggered seismicity. This tool, firstly, computes the background probabilities for all events by eq. (9); then it compares the expected 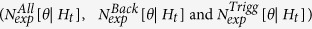 and observed (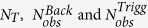) overall, background and triggered target events, by using the equations (11)) and (13); finally it displays the histograms of background and triggered probabilities (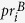 and 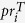).

The *Number of events test* Fig. 4c is a two-tailed test consisting in comparing the observed number of target events *N*_*T*_ with the distribution of events obtained by *NCAT* synthetic catalogs, simulated by using a TM or a TMS ETAS model^21^. It is a revised version of the N-test adopted by the CSEP laboratories^22^, since it differently estimates the p-values of the test. Specifically, the N-test measures the probability to observe *N*_*T*_ target events assuming a Poisson distribution with expectation 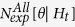 given by the ETAS model under testing. In this way the model gives only information on the expected value 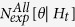, but not on its uncertainty. The probabilities to observe more (*δ*_*1*_) or less (*δ*_*2*_) events than *N*_*T*_ are given by


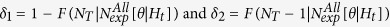


where 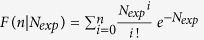
is the Poisson cumulative distribution function with expectation *N*_*exp*_, evaluated at *n*.

The Poissonian hypothesis assumed by the N-test should be unreliable for the model under testing^21^,^23^, so SEDAv1.0 computes both the expectations and the p-values of the test from the ETAS model. The procedure consists of the following steps:

1. Simulation of *NCAT* catalogs, in agreement with the ETAS model under testing;
2. Application of a normal kernel smoothing method to the simulated numbers of events, to obtain a probability density function for the number of target events expected by the model;
3. Computation of the median, 95% and 99% confidence bounds of the inferred distribution;
4. Computation of the probability to observe more than *N*_*T*_ events from the inferred distribution.

When the algorithm terminates, the median expected number of events and the 95% and 99% confidence bounds are reported on the man GUI, together with a plot of the probability density function of the expected number of target events. Finally, the probability 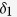 to observe a larger number of events then *N*_*T*_ is computed.

In the section “Examples” we compare the SEDAv1.0 Number of events test with the CSEP N-test, through a tutorial example.

The *Log-Likelihood* (left one-tailed) *test* (Fig. 4d) is a revised version of the L-test adopted by the CSEP laboratories^22^. It consists in computing the probability *ψ* to have lower values of the observed Log-likelihood value, given the model under testing. As the N-test, the L-test computes this probability by assuming a Poisson distribution for each cell, with expectation 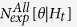
given by the model, and by summing the poissonian log-likelihood for each longitude-latitude bin^22^. Specifically, if 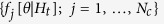
and 
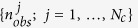
are the forecasts and the observations for all cells *C*_*j*_, the Log-likelihood is computed as


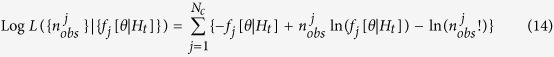


where 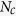 = 1 for TM models.

Then probability *ψ* is computed as the proportion of simulated Log-likelihoods less than the observed Log-likelihood^22^. For the computation of forecast 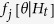, see the next section.

To avoid the Poissonian hypothesis^21^,^23^, SEDAv1.0 adopts a procedure similar to the Number of events test^21^, consisting in the following steps:

1. Simulation of *NCAT* catalogs, in agreement with the ETAS model under testing;
2. Computation of log-likelihoods values on simulated and observed catalogs by eqs. (8);
3. Application of a normal kernel smoothing method to the Log-Likelihood values computed on *NCAT* synthetic catalogs;
4. Computation of the media, 95% and 99% confidence bounds of the inferred distribution;
5. Computation of the probability to observe larger values of the observed Log-Likelihood from the inferred distribution.

When the algorithm terminates, the median expected Log-Likelihood value and the 95% and 99% confidence bounds are reported on the man GUI, together with a plot of the probability density function of Log-Likelihood.

In section “Examples” we compare the SEDAv1.0 Log-likelihood test with the CSEP L-test, by a tutorial example.

#### Forecasting

This tool allows the forecasts calculation by a TM and TMS ETAS model. The forecasts are given in terms of the expected number of events, with magnitude above a threshold *MF* ≥ *Mc*, in the target period [TF1, TF2]. To make forward predictions the target period must follow the interval time [T1, T2] used to estimate the model (see the subsection “Estimation of parameters”).

The forecast calculations are obtained by the following procedure.

1. The system generates *NCAT* synthetic catalogs, covering the target period. To generate these simulations, the system uses the (possible) learning catalog and the TM and TMS ETAS model, given as input. SEDAv1.0 applies the thinning method^2^ generate the simulated catalogs (see the following subsection).
2. The system computes the forecasts for the target period, using the same model adopted in previous step, and the target history of each simulated catalog. The forecasts are computed in terms of expected number of events with magnitude above *MF*, in the target period and (only for a TMS model) in each cell *C*_*j*_ of the background grid. Specifically, the tool computes the forecasts 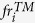 (TM model) and 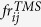 (TMS model), for the i-th simulated catalog, by the formulas

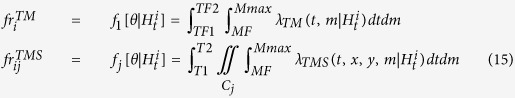


where

1. *MF* ≥ *Mc* is the threshold forecast magnitude;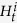 is the history up to time *t*, collected in the *i*-th simulated catalog, including the possible learning events (these last are the same for all simulated catalogs and are collected in the learning catalog, given as input by the operator);
3. if *MF* = *Mc* then 
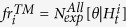
 and 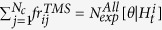.

The representative overall forecast 
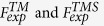
 for the target period [TF1, TF2] are computed as the *median* (50° percentile) of the *NCAT* expected overall number of events of all simulated catalogs


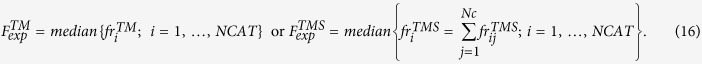


These formulas differ from previous works, in which the forecasts are computed as the *average* of the *NCAT* values 
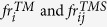
24,25,26,27. The reason behind this choice is that the median is a better estimator for asymmetric and heavy tailed probability distributions, such as those of ETAS forecasts.

The uncertainty about 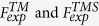
is quantified by the 95% confidence interval of the sets 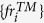 and 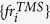.

**Figure 5:**
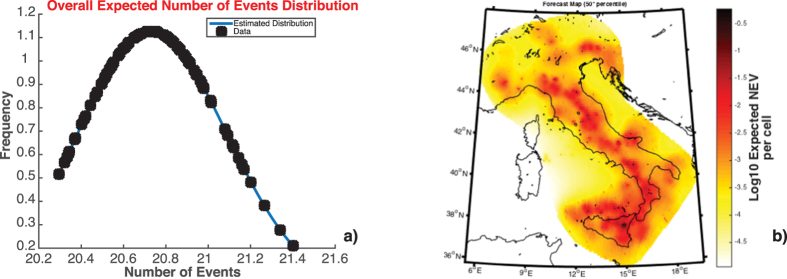
Results for the Forecasts Calculation, based on the estimated ETAS TMS Model on the CSI- 1.1 catalog. The forecast period goes from Jan 01 2003 to Jan 01 2017 and the magnitude threshold is 5.0 (**a**) Distribution of the overall expected number of events in the forecast period, with ML ≥ 5.0. (**b**) Map of the expected number of events with ML ≥ 5.0, in the forecast period, for each cell..

When the algorithm terminates, the system displays some figures and specifically (see Fig. 5):

1. The probability density distribution of the overall expected number of target events above *MF*, obtained by applying a kernel smoothing method on the *NCAT* values 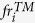 or 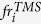 (Fig. 5a);
2. (Only for the TMS models) the maps of a prefixed percentile of the *NCAT* forecasted number of target events 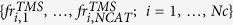 (Fig. 5b);
3. (Only for the TMS models) the histogram of the *NCAT* expected number of target events 
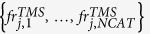
 for each cell *C*_*j*_ of the grid.

The Additional ETAS tool *“Retrospective Forecast”* allows to compare forecasts calculation with the observed seismicity. The system generates the same results and displays the same figures as the ETAS Basic tool *“Forecast”*, with the adding of the comparison with the observations. Since this routine is not a proper test, it was implemented in a module apart from the other tests, among the ETAS additional tools.

#### Simulation

This tool allows the simulation of *NCAT* earthquake catalogs by a TM or a TMS ETAS model. It consists of the combination of two procedures: the thinning method^2^ for the simulations of times and the alias method^28^ to simulate the spatial distribution of background events. The algorithm used to simulate the ETAS catalog is described in Appendix A. Firstly, the algorithm computes the branching ratio


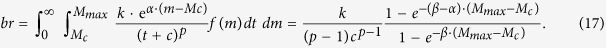


If *br* > 1.0 or *p* < 1.0 the process is explosive, i.e. the number of events in simulations tends toward infinity. In any case, the simulation of each catalog stops when the maximum number (12,000) of events is reached.

When the algorithm terminates all the simulated catalogs are saved in files and a summary of results will appear on the Results Panel and specifically the median number of simulated events, together with the 95% confidence bounds, and the histogram of the number of events in the *NCAT* simulated catalogs, for a magnitude level above *M*_*c*_ chosen by the operator.

The algorithm for ETAS simulations is called also from the tools “Testing the Model” and “Forecasting” of SEDAv1.0 (see previous subsections).

### ETAS Additional Tools

The “ETAS Additional Tools” of SEDAv1.0 are:

1. “*Analysis of Parameters*” and “*Background Map*”: for the retrospective analysis of parameters (including the background spatial distribution) estimated for an ETAS TM or TMS model;
2. “*Trigg/Back Probabilities*”: to compute and visualize the triggering and the background probabilities for an earthquake catalog;
3. “*Identify sequences*”: to identify the sequences in a catalog;
4. “*Retrospective Forecasts*”: to compute a retrospective forecast and to compare observations and expectations.

All the Additional tools, but “*Identify sequences*”, use the output files of an ETAS Basic tool or algorithm previously described (see subsections “Estimation of Parameters”, “Declustering” and “Forecasting”) and do not need further explanation.

### Identify sequences

This tool allows the identification of sequences in an earthquake catalog, by means of a TM and TMS ETAS model. Specifically, this tool consists in applying the stochastic reconstruction, proposed by Zhuang *et al*.^12^, a certain number (*NRUN*) of times. This procedure consists in classifying the events into family trees. In this way, the tool assigns the set of integers {*is*_*i,1*_*, …, is*_*i,NRUN*_} to each event *E*_*i*_, where *is*_*i,k*_ marks the number of the sequence of *E*_*i*_, for the *k*-th run.

In each run *k* and for each event (*T*_*i*_*, X*_*i*_*, Y*_*i*_*, M*_*i*_) the following algorithm is applied

1. a random uniform number u (going from 0 to 1) is compared with the probability 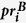 (see eq. (9))
2. if u < 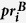 **then**

**the event is assigned to background and** ***is***_***i,k***_** **=** 1**

**else**

**the event is triggered, its parent is the event J** selected as the smallest 0 ≤ *J* < *i* such that


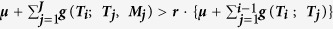
 and is_i,k_ = is_J,k_.

The user of SEDAv1.0 may choose a target event 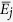 and select all the events belonging to the same sequence of 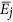, with a probability above a chosen probability level *PL*. The probability *P*_*i*_(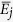) that *E*_*i*_ and 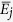 belong to the same sequence is computed as the proportion of times, out of *NRUN, E*_*i*_ belongs to the same sequence of 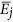, so that


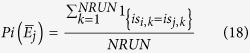


By running the tool more times, the number of events and the temporal limits of the sequences may slightly change, due to the randomness of the method.

At this stage, you may visualize some information, by selecting a probability level *PL* and the target event 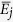. SEDAv1.0 selects all the events *E*_*i*_ with 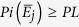 (composing the sequence of the “target event” 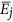) and returns the following results:

1. The times of the first and the last earthquakes and the number of events in the sequence (included the target event);
2. A time-magnitude plot of the events in the sequence;
3. (Only for the TMS model) the map of the events of the sequence.

### Examples

This section illustrates an example of application of most tools of SEDAv1.0 to the Italian seismic catalogue CSI-1.1^29^ (http://csi.rm.ingv.it/versione_inglese/index_eng.htm). It collects 91,797 localized earthquakes, occurred from 1981 to 2002 in Italy.

The SEDAv1.0 “B-value analysis” tool, applied to earthquakes with magnitude ML > 0.0 and depth above 40 km (39544 events), identifies a completeness magnitude ML2.5 from January 01 1991 (5867 events, Fig. 1). The GFT and MBS methods are consistent in estimating a bvalue equal to 0.97, with an error of 0.01.

The ETAS model, implemented in SEDAv1.0, is applied and tested on the selected seismicity. The first step consists in estimating the parameters of the TMS ETAS model, with the learning period January 01 1991, 00:00:00–January 01 1992, 00:00:00 and the testing period January 01 1992, 00:00:00–January 01 2003, 00:00:00. The background grid adopted here is the grid defined for the Collaboratory for the Study of Earthquake Predictability (CSEP) experiment in Italy^30^. It covers the whole national territory, excluding the Sardinia region, and consists of 8993 cells with a side of 0.1°. The SEDAv1.0 “Estimation of parameters” tool (with *NRUN* = 20) provides the set of parameters 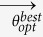 listed in Table 3, together with the 95% confidence bounds. Figure 2 shows some of plots of SEDAv1.0 and specifically the distribution of 
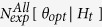
, the values of 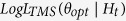 and the map of background probabilities {*u*_*i*_*, i* = *1*, …, *N*_c_} for the best model.

The random declustering procedure, with NCAT = 1000, provides a median number of background events equal to 2349 versus an expected number of events of 2352. The lower and upper 95% confidence bounds are 2309 and 2390, respectively. The numerical and graphical results, given by SEDAv1.0, are shown in Fig. 3.

An example of forecast consists in computing the expected number of events in Italy, above ML5.0, for the forecast period going from January 01 2003 to January 01 2017. By using *NCAT* = 1000 simulations, SEDAv1.0 gives as median number of events 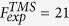. The real number of events occurred is 23. Figure 4 shows the distribution of the overall number of events and the map of the forecast median rates.

Finally, I apply the tests implemented in SEDAv1.0 on the CSI1.1 catalog, to test the ETAS model. Whereas, the KS1 test provides a high p-value (0.5), the RUNS test rejects the model (9·10^−7^). The comparison between the observed and expected number of events (Fig. 5a,b) shows that the inconsistencies between model and data are mainly limited to the occurrence of the 1997–1998 Umbria Marche sequence. The Number of events and the Log-likelihood tests do not reject the model, since the observed values match with the expected distributions (Fig. 5c,d).

To clarify further what was said above about the differences between SEDA1.0 tests and the CSEP procedures, the following shows an application of SEDAv1.0 tests on a TMS ETAS simulated catalog. This collects events with magnitude above 2.5 and covers the Italy region and the time period from Jan-01-2000 to Dec-31-2010. The largest event occurs on April-03-2003 and has a magnitude equal to 6.5. The catalog may be found in the Supplementary Material (file cat_sim.txt).

The TMS ETAS model used for simulating has the parameters *μ* = *0.7, k* = *0.03, p* = *1.2, c* = *0.017, α* = *1.4, d* = *0.7, q* = *1.5, γ* = *0.4* and *b* = *1.0*. The background grid {*u*_*i*_*, i* = *1,N*_*c*_} is attached in the Supplementary Material (file back.txt).

The catalog collects 40 events which occurred between April-04-2003 00:00:00 and April-05-2003 00:00:00, which mainly are aftershocks of the M6.5 event of April-03-2003. The median overall number of expected events by the ETAS model (see eq. 16) is 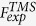 = 22.8. The forecasts 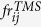 are computed by running the SEDAv1.0 ETAS Basic tool *“Forecasting”* (see subsection 4.1.5), for the period April-04-2003 00:00:00 to April-05-2003 00:00:00, and including all the events that occurred before April-04-2003 as learning. The ETAS model is the same as the one used to simulate the catalog and the number of simulations *NCAT* is equal to 1000.

The expected number of events per cell 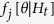, given by the ETAS model, requested to apply the L-test (see eq. 14), are computed after rescaling the forecasts 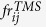
by 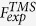. Specifically, since the median of the sum is not the sum of the median, 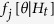, is given by


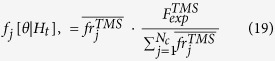


where 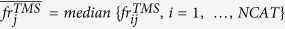, in order to preserve the overall expected number 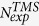
(i.e. 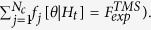


**Figure 6:**
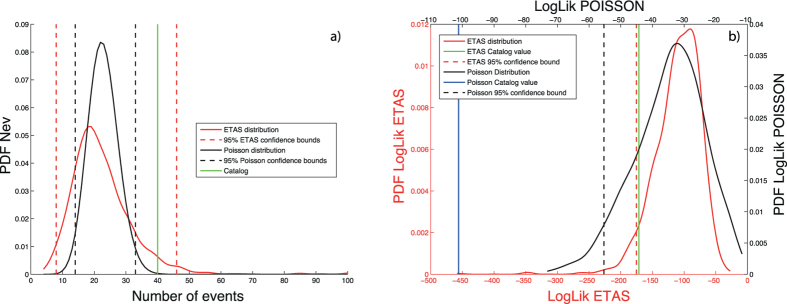
Comparison of the SEDAv1.0 Number of events (panel a) and Log-likelihood (panel b) tests with the CSEP N and L tests, respectively, for a simulated TMS ETAS catalog. The black and red solid lines mark the distributions for the SEDAv1.0 and CSEP tests, respectively. The dotted lines are the limits of the rejection region. In panel (a) the green line marks the number of events of simulated catalog. In panel (b) the green and the blue lines mark the log-likelihood values computed on the ETAS simulated catalog, for the SEDAv1.0 log-likelihood test and CSEP L-test, respectively.

Figure 6 compares the SEDAv1.0 Number of events and Log-likelihood tests with the N and L-tests adopted by CSEP^22^, for the day April-04-2003. Both the Number of events and the Log-likelihood tests are performed with *NCAT* = *1000* simulations. The numbers of events simulated by the ETAS model are reported in the file NTEST_ETAS.txt, included in the Supplementary Material. The observed number of events *N*_*T*_ = *40* is compatible with the distribution obtained by the ETAS model, but not with the Poisson model with rate 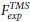, adopted by the N-test procedure, since *N*_*T*_ is well outside the 95% confidence bounds. Similarly, the Log-likelihood of the ETAS model, computed by eq. (8), is in agreement with what is expected from the model, whereas the Log-likelihood of the Poisson model is well below the 95% confidence bound. The simulated ETAS log-likelihoods are reported in the file LTEST_ETAS.txt, included in the Supplementary Material.

## Conclusions

The program SEDA, presented in this paper, has been developed with the main aim of facilitating the user control of the implemented tools, by means of a graphical interface. The core of the first version of SEDA is a set of tools allowing the most important operations related to the ETAS modeling.

From a scientific point of view, one of the most important novelties, with respect to the other free available codes, is the estimation method of the ETAS model on a database, based on the simulated annealing. This ensures the determination of the overall best solution and the evaluation of possible correlations between the parameters^19^. This last feature feature is of prominent importance to detect the multimodality of the log-likelihood function, to attribute a physical significance to parameters and to discuss possible their spatio-temporal variations.

Much consideration has been devoted to the treatment of the background spatial distribution of the ETAS model, which is a point requiring much further investigation. SEDA allows the user both to estimate from the catalog and to fix the background spatial probability distribution {*u*_*i*_*, i* = *1,…,N*_*c*_}. This second option is useful to explore the role of the background in the ETAS modeling or to take into account a priori information from geological or geodetic data.

Particular care has been taken for an effective evaluation of the model uncertainties and for the quantification of their effects on forecast calculations. In SEDAv1.0 there is not an explicit differentiation of the aleatory and epistemic uncertainties, which will be clearer in the following versions. In any case, SEDAv1.0 provides the confidence bounds for all parameters and quantifies the uncertainty about the background spatial distribution. These last come from two sources: firstly, the multimodality of the log-likelihood function, causing possible correlation of parameters, and, second, the inability of data to reveal clearly the basic features of short-term interactions.

An essential part of SEDAv1.0 is devoted to test the ETAS model. Besides the well-known Residual Analysis^1^,^2^, SEDAv1.0 contains two tests: the Number of Events and the Log-likelihood tests. These are a revised version of the N and L tests applied for the CSEP experiments, which are not suitable for time-dependent models^23^. A quantitative evaluation of the spatial performance of the ETAS model and of forecast calculations is still lacking in SEDAv1.0. These points will be implemented in the future, together with further tests.

SEDA has been designed mainly to satisfy a “reproducibility” criterion. Making a research reproducible helps to check the correctness of results and to show the credibility of the science. Moreover, a reproducible research enables others to make use of methods, which are often difficult to recover from published articles, and of results. Finally, the code sharing allows to extend approaches to new applications and to transmit knowledge to future researchers.

Research interests and collaborations will drive the future of SEDA. New technical improvements have been scheduled such as the increase of the maximum catalog size allowed or the addition of a version for Windows systems. Some possible advances will be the implementation of various magnitude distributions and of new testing methodologies, with particular care for the spatial distribution, or some improvements to the ETAS modeling and more specifically to the background estimation and to the quantification of uncertainties.

Great effort has been devoted to test all the tools of SEDAv1.0, but some undetected errors may exist. Suggestions and comments regarding other possible additions to SEDA or bugs to report are always welcome.

## Additional Information

**How to cite this article:** Lombardi, A. M. SEDA: A software package for the Statistical Earthquake Data Analysis. *Sci. Rep.* **7**, 44171; doi: 10.1038/srep44171 (2017).

**Publisher's note:** Springer Nature remains neutral with regard to jurisdictional claims in published maps and institutional affiliations.

## Supplementary Material

Supplementary Information

Supplementary Dataset 1

Supplementary Dataset 2

Supplementary Dataset 3

Supplementary Dataset 4

## Figures and Tables

**Table 1:** Summary of previously published tools on the ETAS modeling. The symbols P and NP mark parallel and non-parallel tools, respectively.

Code-Package and web site	References	Authors	Type of Fortran Routines	Topics	Language
**SASeis2006**	1, 9	Y. Ogata	**NP**	Model Estimation, Simulations, Residuals Analysis	Fortran
http://www.ism.ac.jp/~ogata/Ssg/ssg_softwaresE.html					
**SAPP**					
https://cran.r-project.org/web/packages/SAPP/		M. Saga and J. Nakano	**NP**		R
**PtProcess**	10	D. Harte	**P**	Log-Likelihood Calculation, Conditional Intensity	R
http://cran.at.r-project.org/web/packages/PtProcess					
**ETAS8p**	11, 12	J. Zhuang	**P**	Model Estimation,	Fortran
http://bemlar.ism.ac.jp/zhuang/software.html					
**ETAS**					
http://cran.rproject.org/web/packages/ETAS/index.html		A. Jalilian	**NP**	Declustering, Model Estimation	R
**AftSimulator** http://pasadena.wr.usgs.gov/office/kfelzer/AftSimulator.html	13	K. Felzer	**NP**	Simulations	MATLAB
http://www.geos.ed.ac.uk/homes/stouati	14	S. Touati	**NP**	Simulations, Branching ratio	R
**etasFLP**					
https://cran.r-project.org/web/packages/etasFLP	15	M. Chiodi and G. Adelfio	**NP**	Model Estimation	R

**Table 2:** Summary of tools implemented in SEDAv1.0. The symbols P and NP mark parallel and non-parallel tools, respectively.

Classes	Sub-classes	Tool		Type of Fortran Routine
Catalog analysis		Subset selection		
		B-value analysis		NP
		Figures		
ETAS Model	Basic ETAS Tools	Estimation Parameter		P and NP
		Log-Likelihood Calculation		NP
		Declustering		NP
		Testing the Model	Residual Analysis	NP
			Number of events test	NP
			Log-likelihood Test	P and NP
		Forecasting		P and NP
		Simulation		NP
	Additional ETAS Tools	Analysis of Parameters		
		Background Map		
		Trigg and Back Probabilities		P and NP
		Identify sequences		P and NP
		Retrospective Forecasts		P and NP

**Table 3:** ETAS TMS parameters estimated on CSI 1.1 catalog by using the SEDAv1.0 tool. The 95% confidence bounds are reported in the brackets.

μ	**0.54** (0.53, 0.55)
k	**0.031** (0.030, 0.033)
p	**1.09** (1.08, 1.11)
c	**0.010** (0.009, 0.013)
α	**1.07** (1.02, 1.12)
d	**1.4** (1.3, 1.4)
q	**1.4** (1.39, 1.42)
γ	**0.04** (0.04, 0.10)

## References

[b1] Ogata Y. Statistical models for earthquake occurrences and residual analysis for point processes. J Am Stat Assoc 83, 9–27 (1988).

[b2] Ogata Y. Space-Time Point-Process models for Earthquake Occurrences. Ann. Inst. Statist. Math 50, 379–402 (1998).

[b3] Irving D. A minimum standard for publishing computational results in the weather and climate sciences. B. Am. Meteorol. Soc. 97, 1149–1158, doi: 10.1175/BAMS-D-15-00010.1 (2015).

[b4] Liu L.. Importance of bitwise identical reproducibility in earth system modeling and status report. Geosci. Model Dev. 8, 4375–4400, doi: 10.5194/gmdd-8-4375-2015 (2015).

[b5] Nature Editors. Code share. Papers in Nature journals should make computer code accessible where possible. Nature 514, 536, doi: 10.1038/514536a (2014a). 25355323

[b6] GMD Executive Editors. Editorial: The publication of geoscientific model developments v1.0. Geosci. Model Dev. 6, 1233–1242, doi: 10.5194/gmd-6-1233-2013 (2013).

[b7] McNutt M. Journals unite for reproducibility. Science 346(6210), 679, doi: 10.1126/science.aaa1724 (2014). 25383411

[b8] Nature Editors. Journals unite for reproducibility. Consensus on reporting principles aims to improve quality control in biomedical research and encourage public trust in science. Nature 515, 7, doi: 10.1038/515007a (2014b).

[b9] Ogata Y. *ISM Computer Science Monographs*, No. 33, The Institute of Statistical Mathematics, Tokyo, Japan (2006).

[b10] Harte D. PtProcess: An R Package for Modelling Marked Point Processes Indexed by Time. J Stat Softw 35(8), 1–32 (2010).21603108

[b11] Zhuang J., Ogata Y. & Vere-Jones D. Stochastic declustering of space-time earthquake occurrence. J. Am. Stat. Assoc. 97, 369–380 (2002).

[b12] Zhuang J., Ogata Y. & Vere-Jones D. Analyzing earthquake clustering features by using stochastic reconstruction. J. Geoph. Res.109, B05301, doi: 10.1029/2003JB002879 (2004).

[b13] Felzer K. R. & Brodsky E. E. Decay of aftershock density with distance indicates triggering by dynamic stress. Nature 441, 735–738 (2006). 16760974 10.1038/nature04799

[b14] Touati S., Naylor M., Main I. G. & Christie M. Masking of earthquake triggering behavior by a high background rate and implications for ETAS inversions. J. Geophys. Res. 116, B03304 (2011).

[b15] Adelfio G. & Chiodi M. Alternated estimation in semi-parametric space-time branching- type point processes with application to seismic catalogs. Stoch Environ Res Risk Assess. 29, 443–450, doi: 10.1007/s00477-014-0873-8 (2014).

[b16] Cao A. M. & Gao S. S. Temporal variation of seismic b-values beneath northeastern Japan island arc. Geophy Res Lett 29(9), doi: 10.1029/2001GL013775 (2002).

[b17] Woessner J. & Wiemer S. Assessing the quality of earthquake catalogues: Estimating the magnitude of completeness and its uncertainty. Bull. Seism. Soc. Am. 95, 684–698 (2005).

[b18] Wiemer S. & Wyss M. Minimum magnitude of complete reporting in earthquake catalogs: examples from Alaska, the Western United States, and Japan. Bull. Seism. Soc. Am. 90, 859–869 (2000).

[b19] Lombardi A. M. Estimation of the parameters of ETAS models by Simulated Annealing. Sci Rep. 5, 8417, doi: 10.1038/srep08417 (2015). 25673036 PMC4325320

[b20] Ingber L. Adaptive simulated annealing (asa): Lessons learned. Control Cybern 25(1), 33–54 (1996).

[b21] Lombardi A. M. Some reasoning on the RELM-CSEP Likelihood-Based Tests. Earth. Planets Space 66(4), 1–4 (2014).

[b22] Schorlemmer D., Gerstenberger M. C., Wiemer S., Jackson D. D. & Rhoades D. A. Earthquake Likelihood Model Testing. Seism Res Lett. 78(1), 17–29 (2007).

[b23] Lombardi A. M. & Marzocchi W. The assumption of Poisson Seismic-Rate Variability in CSEP/RELM Experiments. Bull. Seism. Soc. Am. 100, 2293–2300 (2010a).

[b24] Lombardi A. M. & Marzocchi W. The ETAS model for daily forecasting of Italian seismicity in the CSEP experiment. Ann. Geophys. 53, 155–164 (2010b).

[b25] Marzocchi W. & Lombardi A. M. Real-time forecasting following a damaging earthquake. Geoph Res Lett. 36, L21302. doi: 10.1029/2009GL040233 (2009).

[b26] Marzocchi W., Murru M., Lombardi A. M., Falcone G. & Console R. Daily earthquake forecast during the May-June 2012 Emilia earthquakes sequence (Northern Italy). Ann. Geophys 55(4), 561–567, doi: 10.4401/ag-6161 (2012).

[b27] Marzocchi W., Lombardi A. M. & Casarotti E. The Establishment of an Operational Earthquake Forecasting System in Italy. Seism Reaserch Lett. 85, 961–969, doi: 10.1785/0220130219 (2014).

[b28] Walker A. J. An Efficient Method for Generating Discrete Random Variables with General Distributions. ACM T Math Software 3(3), 253–256 (1977).

[b29] Castello B., Selvaggi G., Chiarabba C. & Amato A. CSI Catalogo della sismicita‘ italiana 1981–2002, v. 1.0. INGV-CNT. *Rome*. www.ingv.it/CSI/ (2005).

[b30] Schorlemmer D. . Setting up an earthquake forecast experiment in Italy. Ann. Geophys 53(3), 1–9, doi: 10.4401/ag-4844 (2010).

