# Supplementary material for: SEDA: A software package for the Statistical Earthquake Data Analysis
Source: Sci Rep. 2017 Mar 14;7:44171. doi: 10.1038/srep44171 (PMC5349582; doi:10.1038/srep44171)
Supplement: Supplementary Information [file srep44171-s1.pdf]

Supplementary material of Manuscript

SEDA: A software package for the Statistical Earthquake Data Analysis

Lombardi A.M.

Istituto Nazionale di Geofisica e Vulcanologia, Via di Vigna Murata 605, 00143 Rome, Italy
email: annamaria.lombardi@ingv.it

Appendix A: Algorithm for simulation of ETAS databases.

The following describes the simulation algorithm of a TMS ETAS database $\{T_i, X_i, Y_i, M_i; i>M\}$, implemented in SEDAv1.0, in a time period $[T1, T2]$, after a precursory history $H_{T1}=\{T_i, X_i, Y_i, M_i; i=1,...,M\}$.
The generation of times T_i is done by following the thinning algorithm[21].

Simulation of times T_i

ALGORITHM 1

- set $i=M+1$ and $T=T1$;
- WHILE $(T<T2)$ do
  - generate an uniform random number $r \in [0,1)$ and set $\lambda_1 = \mu + \sum_{T_i<T} g(T;\ Ti, Mi)$;
  - set $dT = -ln(r)/\lambda_1$ and $T=T+dT$;
  - $\lambda_2 = \lambda_1$;
  - WHILE $r > \lambda_2/\lambda_1$ do
    - $\lambda_1 = \lambda_2$;
    - generate an uniform random number $r \in [0,1)$;
    - set $\lambda_2 = \mu + \sum_{T_i<T} g(T; Ti, Mi)$; $dT = -ln(r)/\lambda_1$ and $T = T + dT$;
    - if $T > T2$ STOP;

    END WHILE
  - set $T_i = T$
  - generate (X_i, Y_i); *! only TMS Model*
  - simulate M_i;
  - set i=i+1;

  END WHILE

Simulation of locations (X_i,Y_i)

The algorithm used to simulate the locations of a TMS ETAS catalog is:

ALGORITHM 2

- generate an uniform random number $r \in [0,1)$;
- select the smallest $0 \le J < i$ such that $\mu + \sum_{j=1}^{J} g(T_i\ ;\ T_j, M_j) > r \cdot \{\mu + \sum_{j=1}^{i-1} g(T_i\ ;\ T_j, M_j)\}$;
- if J = 0 then

  $\{X_i, Y_i\}$ = alias $(\{u_j; j=1,...,N_c\})$!simulation for background events

  else

  $\{X_i, Y_i\}$ = loc_trigg $(\{X_J, Y_J\})$!simulation for triggered events

end

Simulation of locations (X_i, Y_i) for background events

The Alias method is adopted here to simulate the background events and can be used for generating a random variable X with a discrete probability distribution $P=\{p_1, p_2, ..., p_K\}$. This method is based on the property that each discrete probability distribution P can be expressed as a equiprobable mixture of K two point distributions, such that

$$p_i = \frac{1}{K}\sum_{l=1}^{K} q_l \; 1_{V_l=i} + (1-q_l)1_{W_l=i}$$

for K pairs of integers $\{(V_1, W_1),...,(V_K, W_K)\}$ and a discrete probability distribution $Q=\{q_1, q_2, ..., q_K\}$.

The probability distribution Q and the pairs of integers are defines as follows.

ALGORITHM 3

- DO i =1,…,K

    q[i] = p[i] · K

    IF (q[i] < 1) THEN L[i] = 1

    IF (q[i] > 1) THEN H[i] = 1

    V(i) = 1

    W(i) = 1

END DO

- DO i =1, … , K

    WHILE (H[i] = 0 and i ≤ K) DO i = i+1

    IF (I = K+1) STOP

    j=1

    WHILE (L[j] = 0 and j ≤ K) DO j = j+1

    W(j)=i

    q[W(j)] = q[W(j)]+q[j]-1

    IF (q[W(j)] ≤1) THEN H[W(j)] = 0

    IF (q[W(j)]<1) THEN L[W(j)] = 1

    L[j] = 0

END DO

The simulation of a random variable X with a discrete probability distribution P is given by

ALGORITHM 4

- generate a random integer variable X uniformly distributed on 1,…,K
- generate an uniform random number $r \in [0,1)$;
- IF r ≤ q(X) THEN

return X

ELSE

return W(X)

END IF

Finally, the epicentral coordinates of background events are defined as follows:

- Identify a cell C_k by applying the Alias method to the probability discrete distribution $\{u_j, j=1,\ldots,N_c\}$
- Simulate the coordinates (X_i, Y_i) from an uniform bivariate distribution in the cell C_k

Simulation of locations (X_i, Y_i) for triggered events

If J > 0 is identified as father (see Algorithm 2), the location (X_i, Y_i) is simulated from the coordinates (X_j, Y_j) as follows. Firstly, the procedure computes the distance (in Kms) ρ between (X_j, Y_j) and (X_i, Y_i) from the probability distribution specified in eq. (2) and having as cumulative probability distribution

$$F(\rho) = \int_0^\rho \int_0^{2\pi} r \cdot \frac{c_{d,q,\gamma}}{\left[r^2 + d^2 \cdot e^{2\gamma(M_j - Mc)}\right]^q} dr\, d\vartheta = 1 - \left[\frac{d^2 \cdot e^{2\gamma(M_j - Mc)}}{\rho^2 + d^2 \cdot e^{2\gamma(M_j - Mc)}}\right]^{q-1}.$$

A random variable Δ can be generated inverting the distribution $F(\rho)$ by the formula

$$\Delta = d \cdot e^{\gamma(M_j - Mc)} \cdot \sqrt{\frac{1 - u^{1/(q-1)}}{u^{1/(q-1)}}}$$

where u is a uniform random number.

The ETAS model of SEDAv1.0 assumes an isotropic distribution for the triggered events, around the epicenter of the triggering events. So that the polar angle θ between (X_j, Y_j) and (X_i, Y_i) is uniformly distributed in [0, 2π).

In summary, the simulation of coordinates (X_i, Y_i) are given by the following algorithm:

ALGORITHM 5

- generate an uniform random number $u \in [0,1)$;
- set Δ by eq.
- set θ by an uniform distribution in [0, 2π);
- compute (X_i, Y_i) by $\begin{cases} X_i' = X_j' + \Delta \cos\vartheta \\ Y_i' = Y_j' + \Delta \sin\vartheta \end{cases}$

where (X'_j, Y'_j) and (X'_i, Y'_i) are the Cartesian coordinates of epicenters (X_j, Y_j) and (X_i, Y_i), respectively.

Supplementary material of Manuscript

SEDA: A software package for the Statistical Earthquake Data Analysis

Lombardi A.M.

Istituto Nazionale di Geofisica e Vulcanologia, Via di Vigna Murata 605, 00143 Rome, Italy
email: annamaria.lombardi@ingv.it

The supplementary material contains following files:

1. **cat_sim.txt:** simulated catalog analyzed in the example (section 5) of the manuscript. The format is

   $$yr \;\; mt \;\; day \;\; hr \;\; min \;\; sec \;\; y \;\; x \;\; dp \;\; m$$

   where [*yr mt day*] is the date of the event; [*hr min sec*] is the occurrence time; [*y x dp*] are the latitude, longitude and depth, *m* is the magnitude.
2. **back.txt:** background probabilities $\{u_i \, , \, i=1,N_c\}$ of the ETAS model used for the example. The format is

   $$lat_j \;\; lon_j \;\; u_j$$

   where [lat_j lon_j] are the latitude and longitude of the j-th cell and u_j is the daily probability to have a background event inside.
3. **NTEST_ETAS.txt:** file with the simulated numbers of events, obtained by the ETAS model.
4. **LTEST_ETAS.txt:** file with the simulated log-likelihoods, obtained by the ETAS model. They are reported in the first column of the file.
